# Stigma around mental health disclosure in medical students

**DOI:** 10.1080/10872981.2020.1774347

**Published:** 2020-06-04

**Authors:** Aqsa Khurram, Dina Abedi, Mohsin Abedi

**Affiliations:** a Medical Education, Barts and the London, School of Medicine and Dentistry, London, UK; b Medical Education, Faculty of Medical Sciences, Newcastle University, London, UK

**Keywords:** Medical education, non-disclosure, mental health, medical students, medical training

Dear Editor,

First and foremost, we would like to commend this article by Fletcher et al. titled ‘*An exploration of medical student attitudes towards disclosure of mental illness*.’ [1]. As senior medical students in the UK, we have found that little information is provided about disclosing mental illness during medical school. Hence, this research paper is intriguing to us as it addresses an issue infrequently discussed. Despite this study being carried out in a single site in New Mexico, we believe it could be applied on a global scale.

Mental wellbeing, alongside physical health, is essential during medical training and as fully qualified physicians. Mental health can be maintained through accessing appropriate treatment as and when required. A key point highlighted in this article was how the lack of engagement with regards to disclosure coupled with the reluctance to seek help can result in unsafe practice [1].

Parallel to prior studies, a self-report methodology can be a useful approach to collect data about individuals’ perception of mental health. Said perception, especially regarding mental wellbeing, can be time dependent [2]. We would like to praise Fletcher et al. in their commitment towards this research by sending the questionnaire, with weekly reminders, to all enrolled students across all years [1]. We believe it would be interesting to see how students’ attitudes towards disclosure may vary at different time points within the same academic year, particularly during final examinations. Anxiety and depression have a higher prevalence rate in students where examinations are imminent, highlighting the importance for research to specify the time of year, alongside academic class, when exploring student mental wellbeing [2].

With regards to the set survey sent out to all participants, especially the final two questions addressing the reasoning behind non-disclosure, we collectively agree that the sample options provided were extensive. They covered a sufficiently large range of reasons why medical students could fear disclosing their condition(s). To strengthen this list, we would like to suggest the addition of the following; *‘the fear of disclosure being on your permanent record for the remainder of your practicing years’* and ‘*the belief that since you have not yet required or received treatment for your mental health in the last five years, it does not need to be disclosed’*. We believe that these are two valid and important justifications, yet different from the stated list, on why one may not have a dire need to declare their mental illness on official records. Additionally, many individual’s mental disturbances go unnoticed. Without a formal diagnosis, some individuals may not accept or perceive their troubles as a mental illness or feel the need to seek treatment [3].

Undoubtedly, there may be a wide array of rationalisations behind non-disclosure, all of which cannot be covered on a predetermined list. Perhaps, for future endeavours, an open-ended question at the end of the survey could prove as a useful tool. Here participants can suggest, in their own words, their justification behind their attitudes. Despite the subjective nature of questioning, this addition may provide an invaluable insight into the trends between medical education and mental health disclosure [4].

Within our own medical schools, we have noticed an increase in student wellbeing support services since the current pandemic has resulted in distant learning. We believe that this should continue throughout the span of medical school globally, not only during these unprecedented times. In turn, the increase in mental health education as part of the curriculum could combat the barriers to acknowledgement of mental health difficulties. Resultantly, this should produce safer practicing physicians, making this publication highly relevant to the current day and age. We are intrigued to see what the pursuance of a longitudinal study, mentioned in the discussion portion of this paper, will bring to this field of research.

Yours sincerely,
